# Cardiovascular Manifestations of Inflammatory Bowel Disease: Pathogenesis, Diagnosis, and Preventive Strategies

**DOI:** 10.1155/2019/3012509

**Published:** 2019-01-13

**Authors:** Diana-Maria Bunu, Cristian-Eugen Timofte, Manuela Ciocoiu, Mariana Floria, Claudia-Cristina Tarniceriu, Oana-Bogdana Barboi, Daniela-Maria Tanase

**Affiliations:** ^1^Department of Cardiology, Institute of Cardiovascular Diseases, Timisoara 300310, Romania; ^2^Department of Radiology, County Emergency Hospital Timisoara, Timisoara 300723, Romania; ^3^Department of Pathophysiology, Faculty of Medicine, “Grigore T. Popa” University of Medicine and Pharmacy, Iasi 700111, Romania; ^4^Department of Internal Medicine, “Grigore T. Popa” University of Medicine and Pharmacy, Iasi 700111, Romania; ^5^3rd Internal Medicine Clinic, “Sf. Spiridon” County Clinical Emergency Hospital Iasi, Iasi, Romania; ^6^Department of Morpho-Functional Sciences I, Discipline of Anatomy, Faculty of Medicine, “Grigore T. Popa” University of Medicine and Pharmacy, Iasi 700111, Romania; ^7^Institute of Gastroenterology and Hepatology-“Sf. Spiridon” County Clinical Emergency Hospital Iasi, Iasi, Romania; ^8^“Grigore T. Popa” University of Medicine and Pharmacy, Iasi 700111, Romania

## Abstract

Inflammatory bowel disease (IBD) refers to a group of chronic inflammatory diseases that targets mainly the gastrointestinal tract. The clinical presentation of IBD includes both gastrointestinal manifestations and extraintestinal manifestations (EIM). The reported cardiovascular manifestations in IBD patients include pericarditis, myocarditis, venous and arterial thromboembolism, arrhythmias, atrioventricular block, heart failure, endocarditis, valvulopathies, and Takayasu arteritis. The aim of this article is to review the available literature about the possible pathogenic mechanisms and determine preventive measures capable of reducing the incidence and severity of the cardiovascular manifestations. In IBD patients, the incidence of cardiovascular manifestations is low, but higher than that in the general population. Therefore, clinicians should pay attention to any new modification that might indicate cardiovascular involvement in IBD patients, and they should consider chronic inflammatory diseases in patients with cardiac conditions without an evident cause. Considering the role of inflammation in the development of cardiovascular manifestations, the management should include prevention of flares and maintenance of remission for as long as possible. Preventive measures should also include active screening and strict control of the cardiovascular risk factors in all IBD patients.

## 1. Introduction

IBD defines a group of chronic inflammatory diseases that targets predominantly the gastrointestinal tract. IBD can manifest itself in two major forms: Crohn's Disease (CD) and Ulcerative Colitis (UC) [1, 2].

The etiopathogenesis of IBD is not yet fully elucidated, but it is known to involve the interaction between four major components: an aberrant immune system, genetic factors, environmental factors, and intestinal microbiota (therefore, the presence of only one component does not cause the onset of IBD) [3]. The inflammatory response is mediated by immune cells (T-helper 1 and T-helper 17 in CD and T-helper 2 in UC), cytokines (Tumor Necrosis Factor-*α* (TNF-*α*), transforming growth factor-*β*, and interleukins- (IL-) 12, IL-17, and IL23), chemokines, reactive oxygen species, neuropeptides, and nonimmune (myeloid, epithelial, mesenchymal, lymphoid, neurogenic, and endothelial) cells [4, 5].

The primary immune response to one or more stimuli induces tissue destruction and proliferation of endothelial and mesenchymal cells, resulting in a secondary immune response that amplifies the already present inflammation and stimulates fibrosis, tissue remodeling, angiogenesis, and lymphangiogenesis [3]. The nonresolving inflammation determines the installation of a vicious cycle of self-sustaining chronic inflammation and maintenance of angiogenesis, fibrosis, and tissue destruction processes [6].

The main manifestations in IBD are intestinal (abdominal pain, mucoid or bloody stool, rectal bleeding, and tenesmus) and systemic (fever, fatigue, loss of appetite, and weight loss) [7]. IBD can also exhibit a wide range of extraintestinal manifestations (IBD-associated disorders that affect organs that are distant to the digestive tract): hepatobiliary, genitourinary, musculoskeletal, respiratory, ophthalmic, cutaneous, and cardiovascular [8, 9].

Cardiovascular manifestations in IBD can be defined by IBD-associated disorders that affect the cardiovascular system [9]. Cardiovascular manifestations in patients with IBD mostly occur as immune-related consequences and include the following: pericarditis, myocarditis, venous and arterial thromboembolism, left ventricle impairment, arrhythmias and conduction disorders, infective endocarditis, valvulopathy, and Takayasu arteritis [10]. The pathogenic mechanisms behind the cardiovascular manifestations are briefly presented in each section and in Table 1.

## 2. Epidemiology of Cardiovascular EIM

Frequencies of EIM in IBD range from 6% to 47%, and multiple EIM may concomitantly occur. Moreover, EIM may occur prior to the diagnosis of IBD in up to 25% of cases [11].

The cardiovascular disease incidence among IBD patients is modestly higher than that in the general population [12]. This fact is supported by a recent Danish cohort study that also observed that the prevalence of traditional cardiovascular risk factors is surprisingly low among IBD patients [13]. Cardiovascular involvement rarely occurs in IBD patients, but its incidence should not be ignored, considering the serious impact of the consequences if untreated.

In 2016, Card et al. published their results on the incidence of EIM in IBD patients. Their study showed a slightly increased diagnosis rate for ischemic heart disease, atrial fibrillation, and hypertension in IBD patients when compared to the general population (odds ratio = 1.85 vs. 0.77, 1.51 vs. 0.56, and 1.69 vs. 0.51, respectively). There were similar diagnosis rates between CD and UC patients [14].

Pericarditis represents the most frequent cardiovascular EIM in IBD patients (70% of the total number of cardiovascular complications) [15, 16]. Its prevalence is 0.19% among the CD patients and 0.23% among the UC patients [17]. A review of 68 patients with IBD showed that pericarditis occurs more frequently in male patients with UC [16].

A 16-year Danish nationwide cohort study of 15572 IBD patients found that myocarditis had a total risk of 4.6 per 100000 years of risk in IBD patients. The incidence rate ratio for myocarditis was 8.3 for CD and 2.6 for UC when compared to the general population [18].

Patients with IBD may present with both venous and arterial thromboembolic complications. They present a 1.7-5.5-fold greater risk for venous thromboembolism than the general population [19]. The Swiss Inflammatory Bowel Disease Cohort Study showed a 3.9 prevalence of VTE among the IBD patients, with a higher incidence among the UC patients when compared to the CD patients (4.7 vs. 3.4, respectively) [20]. Moreover, the mortality associated with thromboembolism is 2 times higher in patients with IBD when compared to the general population [21].

There is an increased risk for arterial thromboembolic events in IBD patients (but to a lesser degree than venous thromboembolism), with similar rates in UC and CD patients [22]. Patients with IBD have a moderately higher risk (18%) of arterial thromboembolic events [23]. The arterial thromboembolic events occur most frequently at the cardiac (acute myocardial infarction), cerebral (stroke), and intestinal (mesenteric ischemia) levels [12]. Their incidence in IBD patients when compared to the general population is 1.2 times higher for acute myocardial infarction, 3.5 times higher for mesenteric ischemia and 1.2 times higher for stroke [22]. In 2013, a Danish cohort study has shown that patients with IBD are at an increased risk of arterial thromboembolism especially during flares and episodes of persistent disease activity [24]. Feng et al. conducted a meta-analysis study on the risk of ischemic heart disease in patients with IBD, and they have observed an increased risk in young female patients with CD [25]. An increased risk of stroke associated with IBD (the risk is greater for UC patients), independent of gender, was found in the meta-analysis conducted by Katsanos et al. [26]. There is also an increased risk of peripheral artery disease (the stenosis in arteries other than those supplying the heart and brain) among IBD patients [27, 28]. Koutroubakis presented in his literature review that most cases with the involvement of the peripheral arteries presented with thrombosis of the upper and lower limbs and they were associated with significantly increased morbidity and mortality [29].

There are not many studies concerning the prevalence of heart failure among the IBD patients. However, a recent population-based cohort study showed a twofold higher risk of heart failure in IBD patients (adjusted hazard ratio, 2.03; 95% CI, 1.36–3.03) and 2.5-fold increased risk in systemic corticosteroid users (adjusted hazard ratio, 2.51; 95% CI, 1.93–4.57) when compared to the control group. Also, it appears that only patients with UC have a significantly higher relative risk of heart failure [30]. Another Danish population cohort study found an increased risk of first heart failure hospitalization in patients with IBD, with a 2.5 times higher risk during the periods of active disease (persistent activity or flares) [31].

The incidence of atrial fibrillation is 11.3% in patients with IBD versus 0.9% in the general population [32]. A Danish cohort study found a 2 times higher risk of atrial fibrillation in IBD during flares and episodes of persistent activity [33]. It is important to acknowledge the increased risk of atrial fibrillation in IBD patients since this arrhythmia is associated with an increased risk of thromboembolism, heart failure, and mortality, it affects the quality of life and exercise capacity, and it increases the hospitalization risk [34].

Concerning the endocarditis prevalence in IBD patients, there are some case reports cited in the literature and a retrospective and prospective case control study from 1992 that shows an increased prevalence of bacterial endocarditis in patients with IBD [35, 36].

There have been several case reports cited in the literature that presented patients with IBD who developed mitral or aortic regurgitation, but its incidence is very rare [37].

The coexistence of IBD and Takayasu arteritis is also rare, with just a small number of reported cases in the literature [38].

## 3. Myocarditis and Pericarditis

Pericarditis represents the most common cardiovascular complication in IBD [13]. Myocarditis can be defined as an inflammation of the myocytes and the interstitial tissue. Occasionally, the pericardium may also be involved, in which case it is called myopericarditis [39].

Patients with IBD have a higher risk for developing myopericarditis than the general population. This can be explained by two mechanisms: autoimmune mediation generated by exposure to autoantigens and drug toxicity following an administration of 5-aminosalicylic acid (5-ASA) or its derivatives [40].

Exposure to autoantigens in an acute episode may cause direct cytotoxicity on myocytes, causing the release of inflammatory mediators and activation of the immune system. This sequence of events can lead to acute myocarditis [41]. If myocarditis is not detected in the acute or subacute stage and the inflammation is not counteracted, myocardial destruction will continue and patients will develop chronic myocarditis [42]. Remodeling processes that are characteristic of chronic inflammation may cause dilation of the cardiac cavities (resulting in systolic dysfunction, anomalies of parietal kinetics, and decreased ejection fraction), valvular regurgitation (by rupture of papillary muscles), or arrhythmias (due to the fibrosis of the conduction system, occurrence of reentry phenomena, and excessive adrenergic stimulation) [43].

The clinical picture for myopericarditis is nonspecific. Patients may present with symptoms similar to those of the acute coronary syndrome, heart failure (new onset or decompensated heart failure), arrhythmias, cardiogenic shock, or sudden death [43]. The occurrence of such clinical picture within the first 28 days since the initiation of the treatment with 5-ASA or its derivatives raises the suspicion of drug toxicity [44].

The 12-lead electrocardiogram may be normal, or it may reveal an ST segment elevation or depression, a negative T wave or rhythm, or conduction disorders [45]. Blood tests may indicate elevated levels of biomarkers of cardiac injury (troponin, creatine kinase, creatine kinase-MB, alanine aminotransferase, and aspartate aminotransferase), as well as B-type natriuretic peptide and N-terminal probrain natriuretic peptide in patients with associated heart failure. Leukocytosis and increased levels of acute-phase reactants (erythrocyte sedimentation rate, C-reactive protein, and fibrinogen) may also be present [46].

Transthoracic echocardiography should be performed in all patients with clinical presentation suggestive for myopericarditis. The presence of left ventricular dysfunction, anomalies of parietal kinetics, low ejection fraction, or accumulation of pericardial fluid should raise the suspicion of myopericarditis [47]. Angiocoronarography should be performed in all patients with the clinical picture suggestive for acute coronary syndrome, since the absence of hemodynamically significant angiographic lesions of the coronary arteries excludes the diagnosis of myocardial infarction [39].

Cardiovascular magnetic resonance (CMR) offers noninvasive characterization of the myocardial tissue, and it can provide the necessary information for the diagnosis of myocarditis. CMR diagnostic criteria for myocarditis reveal myocardial (regional or global) oedema, myocardial hyperaemia, and focal fibrosis or necrosis with noncoronary artery distribution [45]. If patients are hemodynamically stable and CMR is available, then it is recommended to perform CMR before endomyocardial biopsy. Endomyocardial biopsy should be performed in life-threatening conditions, when CMR is not indicated [40].

Endomyocardial biopsy remains the gold standard in diagnosing and establishing the etiology of myocarditis [48]. IBD-associated myocarditis can frequently present under two histopathological forms: acute/chronic lymphocytic myocarditis and giant cell myocarditis (the latter form has a poor prognosis) [43].

Supportive therapy depends on the patients' hemodynamic stability. Patients who are hemodynamically unstable should be redirected to intensive care units that can provide advanced cardiopulmonary support such as mechanical ventilation and extracorporeal membrane oxygenation [46]. Hemodynamically stable patients should be monitored in a hospital setting and treated according to the current guidelines for heart failure with beta-blockers and/or inhibitors of the renin-angiotensin-aldosterone system [49].

Nonsteroidal anti-inflammatory drugs can be used in patients with pericardial involvement [46]. Considering the high possibility of gastrointestinal toxicity, nonsteroidal anti-inflammatory drugs which selectively inhibit the cyclooxygenase-2 should be recommended [50]. Colchicine, another drug usually prescribed for pericarditis, may cause diarrhea as a side effect and, therefore, it can potentially complicate the evolution of IBD [15].

Immunosuppressive therapy may be associated with the supportive therapy, but only after exclusion of infectious etiology. The most widely used immunosuppressive agents are immunoglobulin, corticosteroids, azathioprine, and cyclosporine, and the duration of administration ranges from 3 to 6 months [51].

Management of myocarditis also includes restriction of physical activity during the acute phase and for the following 6 months. Recovery signs include improvement of ejection fraction and parietal kinetics [45].

## 4. Venous Thromboembolism

Deep vein thrombosis and pulmonary thromboembolism represent the most frequent venous thromboembolic complications. But venous thromboembolic events can also occur in the cerebral, portal, mesenteric, or retinal sites [52].

Venous thromboembolism can be triggered by genetic or acquired factors. Long-term hospitalization, surgical interventions, central venous catheters, prolonged bed rest and immobilization, corticosteroid therapy, smoking, use of oral contraceptives, vitamin deficiency, dehydration, hormone replacement therapy, and hyperhomocysteinemia are among the most frequent acquired risk factors for venous thromboembolism [53]. Genetic risk factors such as dysfibrinogenemia, mutations of the prothrombin gene, factor V Leiden, antithrombin, and protein C or S deficiency should be considered in patients with IBD who experience recurrent thromboembolic venous events [54].

Virchow's triad, known to be associated with venous thromboembolism, describes three conditions that predispose to thrombosis: hypercoagulability, endothelial dysfunction, and venous stasis [55].

Hypercoagulability is mediated by the inflammatory process that initiates clotting and interferes with the fibrinolytic system, decreasing the anticoagulant activity [56]. The inflammation in IBD levels can be illustrated by increased levels of C-reactive protein and cytokines (the most frequently observed cytokines are TNF-*α*, vascular endothelial growth factor, and IL-6) [57]. The reduction in anticoagulant activity not only increases thrombosis risk but also helps maintain the inflammatory status by stimulation of thrombin to produce TNF-*α*, IL-6, and IL-10 [58].

In addition, the following hemostatic disorders have been observed during flares: elevated levels of coagulation factors (V, VIII, von Willebrand, and fibrinogen) and products of thrombin and fibrin formation, increased markers of vascular endothelial activation, and acquired deficiencies or dysfunction of natural anticoagulants (protein C, protein S, and antithrombin) [22]. Platelet abnormalities (reactive thrombocytosis, reduced mean platelet volume, and augmented granular content) also contribute to the hypercoagulability. The enhanced activation state of platelets is mediated by the CD40-CD40 ligand pathway [59].

Endothelial dysfunction in IBD patients (procoagulant surface of the vascular bed) is a consequence of mechanical damage (e.g., intravenous catheters) or activation of the vascular endothelial cells by inflammatory mediators. Inflammation determines the occurrence of the thrombophilic effect of the vascular endothelium, and it accentuates the adhesion between the endothelial surface and leukocytes or platelets [22, 60].

The last condition described by Virchow's triad is represented by a disturbance of the blood flow, as it is seen in patients with prolonged immobilization (a common situation encountered during flares in IBD), dehydration, or central vein catheters [22, 61].

The clinical picture of venous thromboembolism depends on the site of the thrombus, and it can range from asymptomatic to severely symptomatic, but the suspicion of venous thromboembolic event is raised when the patient has an unexplained episode of dyspnea, hypoxia or unilateral leg pain, and swelling [62].

The diagnosis of venous thromboembolic events is based on appropriate imaging investigations such as compression ultrasound or venography for deep vein thrombosis, ventilation/perfusion lung scanning or spiral computed tomography for pulmonary emboli, and computed tomography for other affected sites [52, 63].

The primary prevention of venous thromboembolism in acutely ill-hospitalized patients includes the prophylactic administration of one of the following anticoagulants: low-molecular-weight heparin, low-dose-unfractionated heparin, or fondaparinux [64]. Mechanical thromboprophylaxis (graduated compression stockings and pneumatic compression devices) represents a valid alternative for patients who present contraindications for anticoagulation [65].

The primary prevention of venous thromboembolism in hospitalized but stable patients includes prophylactic anticoagulation and management of additional risk factors. Thus, it is intended to maintain the remission, to use intravenous catheters with caution, to correct vitamin deficiencies and dehydration, and to early mobilize the patients [22].

Treatment of acute venous thromboembolism in patients with IBD is similar to that in patients without IBD. The use of anticoagulants (unfractionated heparin and low-molecular-weight heparin) is recommended for mild and moderate venous thrombosis, whereas local or systemic thrombolysis is recommended for massive vein thrombosis [66].

The secondary prevention of venous thromboembolism (anticoagulation) must be individualized according to each patient's hemorrhagic and thromboembolic risks [65]. Long-term anticoagulation using low-molecular-weight heparin, vitamin K antagonists, or novel direct oral anticoagulants is indicated in case of initial unprovoked venous thromboembolic event (in the absence of disease activity or temporary/transient risk factors) [64]. Short-term anticoagulation (3-6 months) is indicated in case of provoked thromboembolic event, and prophylaxis of disease exacerbations can also be added. The placement of inferior vena cava filters is recommended to patients with high thromboembolic risk [66].

## 5. Arterial Thromboembolism

Out of traditional cardiovascular risk factors (male gender, age > 55 years, smoking, obesity, dyslipidemia, arterial hypertension, diabetes mellitus, and chronic kidney disease), advanced age, obesity, and smoking have a reduced prevalence among the IBD patients [67]. But IBD patients also present nontraditional cardiovascular risks: hyperhomocysteinemia, leukocytosis, anemia, corticosteroid therapy, thrombocytosis, high levels of C-reactive protein, and increased erythrocyte sedimentation rate [68].

It is a well-known fact that chronic inflammation and endothelial dysfunction play a role in atherogenesis, one of the most important factors involved in arterial thromboembolism. C-reactive protein, TNF-*α*, vascular endothelial growth factor, and IL-6 represent molecules involved in both atherogenesis and IBD, and their increased serum levels among the IBD patients confirm the fact that atherogenesis is accelerated among this class of patients [57, 69].

There are multiple mechanisms that contribute to the maintenance of the chronic inflammation. IBD patients are also characterized by a disrupted intestinal barrier which allows microbial products (lipopolysaccharides and other endotoxins) to enter the bloodstream. Lipopolysaccharides are known to induce the expression of proinflammatory cytokines, to affect the oxidation of low-density cholesterol, and to activate the macrophages, all of which contributing to endothelial dysfunction, foam cell formation, and atherosclerosis [57].

Obesity (when present) also contributes to the inflammatory status. The adipose tissue is responsible for producing adipokines: leptin, resistin, and adiponectin (proinflammatory cytokines). Mesenteric fat also produces proinflammatory cytokines, such as TNF-*α* and IL-6 [70].

The gut microbiota in IBD is characterized by an abnormal microbial composition (loss of microbial diversity) that can induce immunoregulatory pathways and can mitigate the chronic inflammation [71, 72]. The gut microbiota is also implicated in the atherosclerosis process and increased platelet activation via decreased levels of trimethylamine N-oxide and the induction of expression of Toll-like receptors 2 and 4. Microorganisms can also influence the blood pressure in IBD [73, 74].

Calprotectin is another acute-phase reactant that has been linked to the disease activity in IBD and higher risk for cardiovascular events. Calprotectin binds to Toll-like receptor 4, a receptor that amplifies inflammation and atherosclerosis. Also, calprotectin binds to the receptors for advanced glycation end products (RAGE) which mediate cardiomyocyte dysfunction [75].

The chronic inflammatory process causes structural and functional arterial changes. Smooth muscle cell hyperplasia can be demonstrated by determining carotid intima-media thickness (a subclinical marker of atherosclerosis). Vascular fibrosis and degradation of elastic fibers that occur in the walls of large blood vessels determine vascular stiffness (another subclinical marker of atherosclerosis) [60]. Vascular stiffness is not associated with the cardiovascular risk factors, but with the duration of the episodes of disease activity [76].

Moreover, preexisting coronary atheromatous plaques are more unstable in patients with CD than in the general population. This can be explained by the common genetical characterization of both CD and atherogenesis by a polymorphism of the NOD2/CARD15 gene [77]. Thrombus formation and acute coronary syndrome are favored by the fact that, after the atheromatous plaque has cracked, the lipid core is being exposed to the bloodstream characterized by hypercoagulability and platelet anomalies [58].

IBD patients are also characterized by altered lipid profiles (low levels of total and high-density cholesterol and high levels of low-density cholesterol), known risk factors for atherosclerosis. The exact mechanism is unknown, but it is thought to be either chronic inflammation or malabsorption [78].

Thromboembolic arterial complications also include ventricular thrombosis that occurs, most frequently, in the left ventricle, during flares. Most patients with ventricular thrombosis are asymptomatic, and diagnosis is made predominantly incidental, using imagistic techniques: transthoracic echocardiography (simple or contrast-enhanced), transoesophageal echocardiography, and CMR [79].

The clinical picture depends on the thrombus location, and it can vary from asymptomatic to intensely symptomatic (e.g., chest pain or heart failure symptoms in myocardial infarction and pale, cold, and painful extremities in acute limb ischemia). The probability for arterial thromboembolism is high when the patients complain of chest pain or motor deficits [80–82].

Primary preventive measures of arterial thromboembolism include maintaining the remission, strict control of cardiovascular risk factors, avoiding consumption of oral contraceptives or hormonal replacement therapy, and administration of vitamin B6, B12, and folic acid supplements in case of hyperhomocysteinemia [19]. Acute management and secondary prevention of arterial thromboembolism are not different from those in non-IBD patients [80–82].

## 6. Heart Failure

Acute heart failure can be caused by acute myocardial infarction, myocarditis, pericarditis complicated by tamponade, or endocarditis. Chronic heart failure is caused by valvulopathies, untreated/undiagnosed myocarditis, heart muscle atrophy due to prolonged use of corticosteroids or total parenteral nutrition, and chronic inflammatory status [83–86].

A chronic inflammatory status associated with IBD affects collagen metabolism, causing an inadequate collagen deposit in both affected and distant target organs (demonstrated by elevated serum levels of procollagen III peptides) [84]. This, together with secondary microvascular endothelial dysfunction, alteration of nitric oxide-mediated vasodilation, and deficiencies of vitamins and essential elements, contributes to myocardial fibrosis [85, 86].

Myocardial fibrosis causes left ventricle (LV) impairment: both systolic and diastolic [10]. Transthoracic echocardiography represents the method of choice to diagnose heart failure and evaluate both systolic and diastolic functions of LV. LV ejection fraction reflects the systolic function of the LV, but there are new deformation imaging techniques (strain and strain rate) that can detect subtle abnormalities in the systolic function of the LV even from the preclinical stage [49]. Thus, one can see a low LV global longitudinal strain that can moreover be correlated with the indexes for IBD activity [87–89]. In addition, LV longitudinal strain rates are also reduced, suggesting delayed LV peak contractility [88, 89]. Another sign of LV systolic dysfunction is the detection of abnormalities of wall kinetics (myocarditis and arterial involvement should be taken into consideration) [49].

Diastolic dysfunction can be evidenced in the early stages by measuring the ratio between early mitral inflow velocity and early mitral annular diastolic velocity (one of the most used echocardiographic parameters, a ratio that reflects the LV filling pressures), which will be increased (>14) in patients with IBD with subclinical LV diastolic impairment [90].

## 7. Arrhythmias and Conduction Disorders

Patients with IBD present a predisposition for atrial and ventricular arrhythmias and conduction disturbances [15].

The chronic inflammatory condition found in IBD is the key element in the pathogenesis of arrhythmias. The chronic inflammatory process mediated by proinflammatory cytokines (C-reactive protein, IL-6, and TNF-*α*) causes, through ischemia and oxidative stress, myocardial destruction that, in time, causes interstitial fibrosis and impairs the intracellular calcium current resulting in structural and electrical remodeling, known determinants of arrhythmias [67]. During the active periods of the disease, the enhanced inflammatory status will probably trigger the arrhythmia [34].

Chronic inflammation also leads to the occurrence of autonomic dysregulation (increased sympathetic tone and decreased parasympathetic tone), resulting in reduced heart rate variability and prolonged QT interval, factors that contribute to the development of arrhythmias [92–94]. Heart rate variability in patients with IBD correlates with periods of activity, duration of illness, and inflammatory markers [95].

Patients with IBD experienced increased values for corrected QT interval and corrected QT dispersion. These parameters reflect the ventricular depolarization/repolarization time and conduction heterogeneity at this level. This indicates the increased risk of ventricular arrhythmias in patients with IBD [92, 94]. Obesity, iron deficiency anemia and electrolyte disturbances (hypokalemia, hypocalcaemia, and hypomagnesaemia), and selenium deficiency among IBD patients are additional risk factors for ventricular arrhythmias [16, 94].

P-wave dispersion measured on the electrocardiogram reflects the conduction of the sinus electrical stimulus at the atrial level. Increased values are found in IBD patients, and it indicates the heterogeneity of intra-atrial and interatrial conduction as well as discontinuous propagation of electrical impulses, which predisposes to atrial fibrillation [96]. Intra-atrial and interatrial conduction can also be assessed by the Doppler echocardiography, and IBD patients present an increase in atrial electromechanical delay and a reduction in left atrial mechanical function, changes that correlate with the disease duration (patients with active disease have significantly higher values than patients in remission, but both patients with active disease and in remission have higher values than the general population) [34, 97]. Chronic inflammation also affects the success rate of cardioversion and maintenance of the sinus rhythm in patients with IBD and atrial fibrillation [98].

Atrioventricular conduction disturbances (complete atrioventricular block, second-degree or first-degree atrioventricular block) have been reported in patients with IBD and may occur due to the administration of infliximab, ischemia in the conduction system secondary to inflammation, vasculitis, or microvascular endothelial dysfunction [99–101]. Pacemaker implantation is the gold standard treatment in the complete atrioventricular block and other symptomatic conduction disorders that affect the general status of the IBD patient (syncope, altered general status, fatigue, and worsening of the heart failure) [102].

Careful ECG monitoring (including 24 h Holter ECG), regular determination of serum electrolyte levels, and maintenance of remission for as long as possible are additional measures that need to be taken into consideration in the management of IBD.

## 8. Endocarditis

Cases of infectious endocarditis are reported in the literature in patients with IBD. Predisposing risk factors include immunosuppressive medication, the presence of central venous catheters, and significant preexisting valvulopathies [16]. In addition, IBD patients have an increased risk of secondary bacteremia due to increased transmucosal permeability and secondary immunosuppression due to the corticosteroids or other immunosuppressants [103, 104]. Microbial agents cited in the literature as being involved in the etiopathogenesis of infectious endocarditis are *Enterococcus faecalis*, *Enterococcus faecium*, *Peptostreptococcus*, *Streptococcus bovis*, *Candida albicans*, *and Bacteroides fragilis* [36, 105].

There has been reported a case of nonbacterial thrombotic endocarditis (Libman-Sacks endocarditis) in a patient with IBD. The noninfective endocarditis is associated with a higher thromboembolic risk, especially in IBD patients who are characterized by hypercoagulability [106].

Symptoms and signs that suggest the possibility of endocarditis are fever, heart murmurs, or embolic phenomena. Treatment of infectious endocarditis in patients with IBD does not differ from that in patients without IBD [107].

The Advisory Group of the British Cardiac Society Clinical Practice Committee and Royal College of Physicians Clinical Effectiveness and Evaluation Unit include the IBD patients in the high-risk group. Antibiotic prophylaxis should be mandatory in the case of invasive procedures or central venous catheters, especially if the patient has a preexistent valvulopathy. Furthermore, the use of immunosuppressive therapy and corticosteroids should be also minimized [108, 109].

## 9. Valvulopathies

The most common IBD-related valvulopathies are aortic regurgitation and mitral regurgitation [37].

In IBD, inflammation causes mitral and aortic valvulopathies (where blood pressures are high), and excess TNF-*α* causes the thickening and shortening of the leaflets, resulting in regurgitation [110]. In addition, there is an overwhelming fibroblastic healing phenomenon (seen echocardiographically as a subaortic bump) and a thickening of the aortic intima, both conditions leading to an ascending aortic aneurysm. Thus, localized lesions of the aortic root also contribute to the occurrence of aortic regurgitation [111]. Another possible mechanism is the myxomatous degeneration (collagen deposition on the valve) resulting in a benign valve prolapse or even mild regurgitation [10].

Other changes that may be secondary to the chronic inflammatory process include aortic aneurysm or ectasia, coronary ostial stenosis, and atrioventricular conduction disorders [112].

Early detection of these valvular changes could lead to prevention of flares in IBD or worsening of the valvulopathies [113].

## 10. Takayasu Arteritis

Takayasu arteritis is an autoimmune condition that targets large vessel components. The inflammation of the vessels determines fibrosis, stenosis, and thrombosis [114].

Takayasu arteritis and IBD (especially UC) have several common types of HLA: class I (HLA-A∗24:02 and HLA-B∗52:01) and class II (HLA-DRB-1∗15:02), which could explain their coexistence in some patients with IBD [115].

In patients with IBD and Takayasu arteritis, IBD symptoms are the first to appear. At the same time, in these patients, Takayasu arteritis manifestations appear more rapidly when compared to patients with only Takayasu arteritis, without IBD [116].

The clinical picture includes fatigue, fever, and focal symptoms (depending on the affected vessel): cervical, maxillary, brachial, humeroscapular, or chest pain, whether or not accompanied by unilateral or bilateral paraesthesia [117]. In patients with IBD and Takayasu arteritis, the clinical picture is formed more frequently by constitutional symptoms, headache, vertigo, and gastrointestinal symptoms [118].

Early diagnosis of Takayasu arteritis by means of a thorough clinical examination (vascular murmurs, pulse reduction, and hypertension) and noninvasive imaging methods (transthoracic echocardiography, computed tomography, magnetic resonance imaging, and fluoro-D-glucose positron emission tomography) and early therapy contribute to the prevention of Takayasu arteritis complications: aortic regurgitation, congestive heart failure, renal hypertension, or stroke [38, 116].

Takayasu arteritis treatment includes first-line corticosteroids. If corticosteroids prove to be ineffective or withdrawal is not possible, immunosuppressants will be added: methotrexate, cyclophosphamide, azathioprine, or tacrolimus [117]. Biological agents such as infliximab, rituximab, and tocilizumab can also be used in refractory cases, with infliximab being elective in patients with IBD [114]. Cases of arterial stenosis or occlusion may require surgery or endovascular surgery [117].

## 11. Medicines in IBD Treatment and Cardiovascular Complications

Pharmacological treatment in IBD includes five main classes: aminosalicylates (5-aminosalicylic acid-based compounds), corticosteroids, immunosuppressants (azathioprine, mercaptopurines, and methotrexate), calcineurin inhibitor (cyclosporine), and monoclonal antibodies (infliximab, adalimumab, and certolizumab) [119]. The most frequent cardiovascular side effects are presented in Table 2.

5-ASA and its derivatives represent one of the most widely used drug classes in the treatment of IBD (especially for UC). But myopericarditis is among the side effects of these drugs. Four possible mechanisms can explain the occurrence of these side effects: direct cytotoxicity, cell-mediated hypersensitivity reaction, autoimmune response, or immunoglobulin E-mediated allergic reaction. The first therapeutic measure in myopericarditis secondary to 5-ASA or its derivative is to discontinue the administration [44]. If the symptoms persist, then corticosteroids will be administrated and other causes for myopericarditis will be sought [120]. Rechallenge with an alternative agent may be attempted only after the patient has been hemodynamically stabilized (e.g., mesalazine may be attempted in patients with myopericarditis caused by sulfasalazine) and only under close medical supervision due to the possibility of recurrence of symptomatology [44]. Sinus bradycardia has been associated with mesalamine administration [121].

The most important side effects of corticosteroids affecting the cardiovascular system are hypertension, dyslipidemia, and thromboembolism, all of which contribute to the acceleration of atherosclerosis and increase the risk of acute coronary syndrome [122]. Other side effects are oedema, worsening heart failure, and electrolyte imbalances (hypernatremia and hypokalemia) [123]. Preventive measures to lower the side effect incidence include a diet based on low salt and sugar intake, potassium supplementation, and initiation of antihypertensive treatment in case of hypertension with careful electrolyte monitoring [124]. The contribution of corticosteroid therapy to the reduction of cardiovascular risk is controversial. Although corticotherapy reduces the inflammation, it is associated with a 2.5 higher risk of cardiovascular events, especially because its long-term side effects (diabetes, hypertension, and obesity) represent well-known cardiovascular risk factors [103, 125, 126].

It is worth mentioning that in the literature, there have been reported cases of atrial fibrillation and prolonged QT interval that occurred during azathioprine use (but other risk factors were also present) [92, 127–129]. Other rare cardiovascular side effects of azathioprine are angina, hypotension, venous thrombosis, and cardiogenic shock [130]. Methotrexate administration is associated with hypotension, acute coronary syndrome, pericarditis, myocarditis, thromboembolic events, and methotrexate side effects [131].

Cyclosporine is associated with increased risk of hypertension, arrhythmias, acute coronary syndrome, and heart failure [132].

Biological molecules are associated with increased arrhythmogenic risks: supraventricular tachycardia, atrial fibrillation, ventricular tachycardia, multiple ectopic ventricular beats, bradyarrhythmias, and conduction disturbances [132]. Besides the arrhythmogenic risks, cardiovascular adverse effects include hypotension, hypertension, acute coronary syndrome, dyslipidemia, and worsening heart failure. An important contraindication to infliximab is heart failure class NYHA III and IV [133, 134].

On the other hand, IBD treatment can positively influence the cardiovascular system. For instance, long-term treatment with immunosuppressants may reduce the aortic wall stiffness and improve the endothelial dysfunction, but it does not influence the peripheral arteries [135]. TNF-*α* antagonists increase the cardiovascular risk in patients with IBD, while, surprisingly, thiopurine administration and surgical interventions could, potentially, decrease the risk [136]. 5-ASA treatment reduces the inflammation status, thereby decreasing the cardiovascular risk [137].

## 12. Discussion

During the past two decades, there has been growing evidence that IBD patients can have cardiovascular manifestations besides other EIM. Even though the increased risk of venous thromboembolism in IBD patients has been proven, the prevalence of the rest of cardiovascular EIM is low.

There are studies that deny the moderate risk of arterial thromboembolic events. For instance, Sridhar et al. performed a cross-sectional study to observe the association of different cardiovascular conditions with IBD. They found a significantly increased risk for mesenteric ischemia (adjusted odds ratio 3.4; 95% CI, 2.9-4.0), venous thromboembolism (adjusted odds ratio, 1.38; 95% CI, 1.25-1.53), and arrhythmias (among young females, adjusted odds ratio, 2.05; 95% CI, 1.72-2.44), but not with any other cardiovascular conditions (such as stroke or acute coronary syndrome) [93]. But cardiovascular mortality is not increased in IBD patients when compared to the general population, with no differences between UC and CD [138].

It is difficult to differentiate between true cardiovascular EIM and cardiovascular complications. Considering the rarity of cardiovascular EIM and the large number of side effects associated with the treatment in IBD, when patients present with cardiovascular symptoms and signs, clinicians should try to exclude the possibility of side effects associated with certain drugs used for treating IBD before establishing the disease activity as a causal factor.

Preventive measures that aim to reduce the incidence of cardiovascular EIM and its impact on the disease natural evolution are briefly presented in Table 3.

## 13. Conclusions

Cardiovascular manifestations in patients with IBD, although rare, are characterized by a higher incidence when compared to the general population, and they occur due to the effects of chronic or acute inflammation, drug toxicity, or genetic predisposition. Early recognition helps prevent complications and minimize the impact on the disease's natural course. Among the primary preventive measures for cardiovascular manifestations, some of the most important are maintenance of remission for as long as possible, cardiologic periodic evaluation (physical exam, blood tests, electrocardiogram, and transthoracic echocardiography), anticoagulation of patients with high thromboembolic risk, and management of traditional and nontraditional cardiovascular risk factors.

## Figures and Tables

**Table 1:** Cardiovascular manifestations in IBD.

Cardiovascular manifestations	Possible pathogenic mechanisms	References
Pericarditis and myocarditis	(i) Immune-mediated myocarditis in IBD as a result of exposure to autoantigens	[40–43]
	(ii) Cardiotoxicity as an adverse effect of the treatment with 5-ASA and its derivatives	

Venous thromboembolism	(i) Hypercoagulability induced by the systemic inflammation	[22, 53–61]
	(ii) Platelet abnormalities	
	(iii) Endothelial dysfunction induced by mechanical and systemic factors	
	(iv) Venous stasis	
	(v) Acquired risk factors (prolonged hospitalization, surgical interventions, central venous catheters, prolonged immobilization and bed rest, glucocorticoids, smoking, oral contraceptives, vitamin deficiencies, dehydration, hormone replacement therapy, and hyperhomocysteinemia)	
	(vi) Genetic risk factors (dysfibrinogenemias, prothrombin gene mutation, factor V Leiden thrombophilia, and deficiency of proteins C, S, and antithrombin)	

Arterial thromboembolism	(i) Structural and functional vascular alterations induced by chronic systemic inflammation	[57, 68–78]
	(ii) Accelerated development of atherosclerosis and highly unstable atherosclerotic plaques	
	(iii) Endothelial dysfunction induced by microbial lipopolysaccharides	
	(iv) Altered gut microbiota	
	(v) Adipokines	
	(vi) Calprotectin	
	(vii) NOD2/CARD15 gene polymorphism	
	(viii) Dyslipidemia	

Heart failure	(i) Myocardial fibrosis secondary to altered collagen metabolism, impaired nitric oxide-mediated vasodilation, and deficiencies of vitamins and essential trace elements	[83–87]
	(ii) Heart muscle atrophy due to prolonged use of corticosteroids, total parenteral nutrition, and chronic inflammatory status	
	(iii) Myocarditis, endocarditis, and valvulopathy	

Arrhythmias and conduction disorders	(i) Interstitial fibrosis and structural and functional cardiac remodeling	[91–95, 99–101]
	(ii) Impaired autonomic nervous system: increased sympathetic and decreased parasympathetic activity	

Endocarditis	(i) Bacteremia due to increased transmucosal permeability	[16, 103–106]
	(ii) Predisposing risk factors: immunosuppression, preexistent valvular heart disease, and central venous catheters	

Valvulopathies	(i) Myxomatous degeneration	[110, 111]
	(ii) Ascending aorta changes due to chronic systemic inflammation	

Takayasu arteritis	(i) Genetic risk factors: HLA-A∗24:02, HLA-B∗52:01, and HLA-DRB-1∗15:02	[114, 115]

**Table 2:** Cardiovascular side effects of IBD treatment [44, 92, 120–134].

(i) Hypertension
(ii) Dyslipidemia
(iii) Accelerated atherosclerosis and acute coronary syndromes
(iv) Thromboembolism
(v) Worsening heart failure
(vi) Arrhythmias
(vii) Pericarditis and myocarditis

**Table 3:** Recommended preventive measures for cardiovascular EIM in IBD.

General measures	
(i) Maintenance of remission for as long as possible	
(ii) Regular cardiologic checkup	
(a) Physical examination	
(b) Blood pressure measurement	
(c) Blood tests (hemoleucogram, lipids, electrolytes, biomarkers of cardiac injury, B-type natriuretic peptide, and N-terminal probrain natriuretic peptide, acute phase reactants)	
(d) 12-lead electrocardiogram	
(e) Transthoracic echocardiography (including 2D speckle tracking for early detection of subclinical changes in cardiac function)	
(iii) Reduce stress	
(iv) Cease smoking	
(v) Lose weight in case of obesity	
(vi) Reduce hospitalization	
(vii) Early mobilize the patients	
(viii) Minimize the use of invasive devices	
(ix) Reduce the consumption of oral contraceptives and hormone replacement therapy	
(x) Minimize the duration of corticosteroid and immunosuppressant administration	
(xi) Diet with low sodium intake and potassium supplementation while corticosteroid use	

Specific measures	
Venous thromboembolism [22, 59, 64–66]	(i) Screening for genetic risk factors in patients with recurrent venous thromboembolic events
	(ii) Prophylactic anticoagulation or mechanical thromboprophylaxis (when anticoagulation is contraindicated) and management of additional risk factors in hospitalized patients
	(iii) Short-term or long-term anticoagulation should be decided according to each patient
Arterial thromboembolism [19, 80–82]	(i) Management of traditional and nontraditional cardiovascular risk factors
Infective endocarditis [108, 109]	(i) Prophylactic antibiotherapy in case of preexisting valvulopathy, invasive procedures, or use of central venous catheters
